# An unusual manifestation of hyperkalemia

**DOI:** 10.11604/pamj.2014.19.251.5007

**Published:** 2014-11-07

**Authors:** Ahmed Belkouch, Lahcen Belyamani

**Affiliations:** 1 Emergency Department, Military Hospital of Instruction Mohamed V, Rabat, Morocco

**Keywords:** Hyperkalemia, atrioventricular bloc, emergency

## Image in medicine

An 82 years old patient was admitted to the emergency department complaining since 15 days of fatigue, dizziness and lipothymia. He had a history of hypertension and diabetes; and had undergone a coronary bypass in 2005. His physical examination showed a blood pressure at 160/50mmHg, a heart rate at 26cycles/min, and he was afebrile. On admission the ECG showed a third degree atrioventricular bloc with a regular rhythm at 26 cycles/min, P wave was absent, QRS complex was not wide (<0.10ms) and the T wave was not “tente like”, tall, peaked and symmetric, it was negative in the inferior territory and the ST segment was underlined in the laterobasal derivations. The laboratory findings included a serum potassium level of 6.9mEq/l (normal 3.6-5.5), sodium 132mEq/l (normal 135-140), creatinine 30mg/l (6-14) and urea nitrogen 0.82g/l (0.17-0.43), MDRD clearance was at 20ml/min (normal > 80). The arterial blood gas analysis revealed a pH of 7.32, pO2=115mmHg, and pCO2=34mmHg. The patient was treated immediately with furosemide, bicarbonate serum, calcium gluconate, glucose solution, and insulin, after three hours, the ECG showed normal sinus rhythm with visible P waves, a heart rate at 60cycles/min and a shortening of the QT interval in comparison with the first ECG. Blood potassium at this moment was at 5.60mEq/l. Hyperkalemia is known to cause a depression of the conduction velocity and excitability of the pacemaker cells and conduction tissues, resulting generaly in an advanced atrioventricular bloc with wide QRS complex. In this case the QRS remained normal.

**Figure 1:**
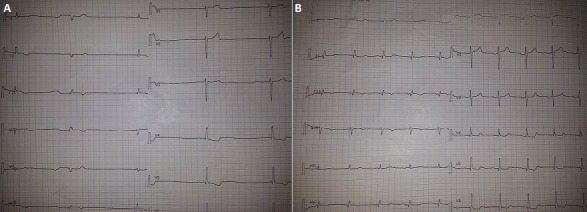
(A) ECG of admission showing the third degree atriventricular bloc, with absence of P wave and normal QRS, (B)ECG after correction of the hyperkalaemia

